# Avian Influenza (H5N1) Virus in Waterfowl and Chickens, Central China

**DOI:** 10.3201/eid1305.061209

**Published:** 2007-05

**Authors:** Zhengjun Yu, Yunfeng Song, Hongbo Zhou, Xiaojuan Xu, Qiaoyun Hu, Haiya Wu, Anding Zhang, Yanjun Zhou, Jianfeng Chen, Hanbing Dan, Qingping Luo, Xiangmin Li, Huanchun Chen, Meilin Jin

**Affiliations:** * Huazhong Agricultural University, Wuhan, People’s Republic of China; 1 These authors contributed equally to this article.

**Keywords:** Avian influenza (H5N1), waterfowl, chickens, People’s Republic of China, dispatch

## Abstract

In 2004, 3 and 4 strains of avian influenza virus (subtype H5N1) were isolated from waterfowl and chickens, respectively, in central People’s Republic of China. Viral replication and pathogenicity were evaluated in chickens, quails, pigeons, and mice. We analyzed the sequences of the hemagglutinin and neuraminidase genes of the isolates and found broad diversity among them.

Several avian influenza outbreaks occurred in Asia during 2003–2004. We performed virus isolation in central People’s Republic of China in early (January–March) 2004 by injecting infected bird tissue homogenates into 10-day-old specific-pathogen–free embryonated chicken eggs, according to standard procedures (*1*). Seven strains of avian influenza (H5N1) virus were isolated and named A/widgeon/Hubei/EWHC/2004 (EWHC), A/chicken/Hubei/327/2004 (CKDW), A/chicken/Hubei/JZJ/2004 (CKJZ), A/chicken/Hubei/TMJ/2004 (CKTM), A/chicken/Hubei/XFJ/2004 (CKXF), A/goose/Hubei/ZFE/2004 (GOZF), and A/duck/Hubei/XFY/2004 (DKXF). During the outbreak, CKDW, CKJZ, CKTM, and CKXF were isolated from free-range chickens in villages. GOZF, which was responsible for 50% of the incidence and 30% of the related deaths of infected geese, was isolated from a farmed goose in a flock of >1,000. EWHC was isolated from a Eurasian widgeon in a large lake where many widgeons were found dead. DKXF was isolated from an asymptomatic domestic duck on a farm where ducks sporadically died.

The isolates were characterized in Madin-Darby canine kidney (MDCK) cells, embryonated eggs, chickens, and mice. The 50% tissue culture infectious dose (TCID_50_), 50% egg infectious dose (EID_50_), intravenous pathogenicity index (IVPI), intracerebral pathogenicity index (ICPI), and 50% lethal dose in mice (MLD_50_) were determined in the respective models. For MLD_50_ determination, mice (n = 5 per dose) were intranasally inoculated with serial dilutions of virus at 10^1^–10^6^ EID_50_ in a volume of 50 μL. TCID_50_, EID_50_, and MLD_50_ were calculated by using the Reed-Muench method (*2*). IVPI and ICPI were determined as previously described (*3*). All experiments were performed in a Biosafety Level 3 laboratory. Related data are summarized in Table 1.

**Table 1:** Characteristics of the 7 avian influenza (H5N1) isolates obtained in central People’s Republic of China, 2004*

	HA titers	Avian				Mammalian		
		EID_50_	IVPI	ICPI	Pathogenicity in chickens†	TCID_50_	MLD_50_	Pathotype in mice‡
EWHC	2^10^	10^7.5^	3.0	1.91	High	10^4.41^	10^1.3^	High
CKDW	2^8^	10^8.23^	3.0	1.78	High	10^7.8^	10^2.3^	High
GOZF	2^8^	10^9.8^	2.98	1.88	High	10^7.35^	10^1.5^	High
CKXF	2^8^	10^6.22^	2.96	1.88	High	10^4.67^	10^6^	Medium
CKJZ	2^8^	10^6.33^	3.0	1.9	High	10^5.24^	>10^6.5^	Low
CKTM	2^8^	10^7.23^	1.71	1.36	High	10^2.0^	>10^6.5^	Low
DKXF	2^8^	10^6.67^	0	1.25	Low	10^6.57^	>10^6.5^	None

*HA, hemagglutinin; EID_50_, 50% egg infectious dose; IVPI, intravenous pathogenicity index; ICPI, intracerebral pathogenicity index; TCID_50_, 50% tissue culture infectious dose;; MLD_50_, 50% lethal dose in mice; EWHC, A/widgeon/Hubei/EWHC/2004; CKDW, A/chicken/Hubei/327/2004; GOZF, A/goose/Hubei/ZFE/2004; CKXF, A/chicken/Hubei/XFJ/2004; CKJZ, A/chicken/Hubei/JZJ/2004; CKTM, A/chicken/Hubei/TMJ/2004; DKXF, A/duck/Hubei/XFY/2004.  †Avian influenza virus isolates are considered to be highly pathogenic if they cause a death rate of >75% within 10 days or have an IVPI of >1.2 (World Organization for Animal Health criteria). ‡The pathotypes in mice were divided into none, low (MLD_50_>6.5 log_10_EID_50_), medium (3.0 log_10_EID_50_<MLD_50_≤6.5 log_10_EID_50_), and high (MLD_50_≤3.0 log_10_EID_50_).

Pathogenicity of the 7 isolates was evaluated in birds. Six-week-old White Leghorn (WL) chickens, 4-week-old quails, and 4-week-old pigeons, 64 each, all free from avian influenza (H5N1) virus infection, were divided into 8 groups of 8 birds. For each species, 7 groups were intravenously inoculated with 10^6^ EID_50_ of 1 of 7 avian influenza (H5N1) isolates, and 1 group received allantoic fluid as a negative control. Birds were monitored daily, and the number of deaths was recorded until 10 d postinfection, at which point all surviving birds were killed. Viral tissue tropism was analyzed in dead (on the day of death) and surviving birds (10 d postinfection) by a double-antibody sandwich ELISA for the nucleoprotein of influenza A virus (*4*). The mean time to death (MDT) was calculated (Table 2).

**Table 2:** Death rates and mean time to death (MDT) from avian influenza virus (H5N1) infections in 3 types of birds inoculated with the 7 isolates obtained in central People’s Republic of China, 2004*

Isolate	Chicken		Quail		Pigeon	
No. deaths/no. inoculated	MDT (days)	No. deaths/no. inoculated	MDT (days)	No. deaths/no. inoculated	MDT (days)
EWHC	8/8	1	8/8	2.8	3/8	4.3
CKDW	8/8	1	8/8	3.4	2/8	5
GOZF	8/8	1.3	8/8	3.3	2/8	5
CKXF	8/8	1.4	5/8	4	0/8	–
CKJZ	8/8	1.1	8/8	3.4	1/8	6
CKTM	6/8	4.7	3/8	6.3	0/8	–
DKXF	0/8	–	0/8	–	0/8	–

* EWHC, A/widgeon/Hubei/EWHC/2004; CKDW, A/chicken/Hubei/327/2004; GOZF, A/goose/Hubei/ZFE/2004; CKXF, A/chicken/Hubei/XFJ/2004; CKJZ, A/chicken/Hubei/JZJ/2004; CKTM, A/chicken/Hubei/TMJ/2004; DKXF, A/duck/Hubei/XFY/2004. Dashes represent no deaths.

All chickens injected with the CKDW or EWHC isolates died within 24 h (MDT<1.0); those injected with GOZF, CKXF, or CKJZ died within 2 d (MDT<1.5). CKTM caused a 75% death rate for chickens, with an MDT of 4.7; all chickens injected with DKXF survived. Quails were also susceptible to infection, but the MDTs in quail (2.8–6.3) were higher than those in chickens. CKDW, EWHC, GOZF, and CKJZ caused a 100% death rate; CKXF and CKTM caused 62.5% and 37.5% death rates, respectively; and DKXF did not cause death. Pigeons were more resistant to these isolates than the other birds; the highest death rate was 37.5%, for the EWHC group, with an MDT of 4.3. CKDW and GOZF caused a 25% death rate with an MDT of 5.0; CKJZ caused a 12.5% death rate with an MDT of 6.0. CKTM, CKXF, and DKXF exhibited low pathogenicity in pigeons, with no deaths by day 10.

Most chicken tissue samples had positive ELISA results for avian influenza (H5N1) infection; no particular tissue tropism pattern was found in quail samples. In contrast, all tested pigeon glandular stomach samples from the 7 infected groups had positive results, while most other organs had negative results.

The pathogenicity of the 7 isolates was also evaluated in mice. Forty-eight 6-week-old female BALB/c mice (6 in each of 8 groups) were anesthetized with dry ice and intranasally inoculated with 10^6^ EID_50_ of 1 the isolates in a volume of 50 μL. One group was inoculated with normal allantoic fluid as a negative control. The mice were monitored daily for clinical signs and death. The surviving mice were killed at 14 d postinfection for the tissue tropism study. EWHC, CKDW, and GOZF caused lethargy, weight loss, lymphopenia, neurologic disorders, and death within 4–13 d. Mice in the EWHC group showed more rapid and obvious weight loss than those in the CKDW and GOZF groups. CKXF caused moderate clinical signs and a 50% death rate; DKXF, CKTM, and CKJZ caused no obvious clinical signs and no deaths (Figure 1). The virus recovery rates of the 7 isolates showed that the lung had the highest recovery rate (19/42) and the heart had the lowest (3/42); they indicated that the virus could be isolated from the brain (17/42), kidney (13/42), liver (11/42), and spleen (7/42). EWHC showed the highest recovery rate (25/36), and DKXF had the lowest (4/36) among the isolates. Despite no obvious clinical signs in mice injected with DKXF, CKTM, and CKJZ, virus could be reisolated from the tissues; the rates of recovery were 6/36 (CKJZ), 6/36 (CKTM), and 4/36 (DKXF). These results indicated that the isolates DKXF, CKTM, and CKJZ could replicate in mice but at a low efficiency.

**Figure 1:**
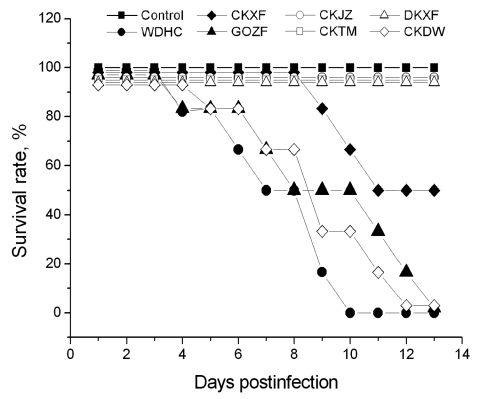
Survival times of mice infected with the 7 avian influenza virus (H5N1) isolates. Mice were intranasally inoculated with 10^6^ 50% egg infectious dose of viruses in a volume of 50 μL. A/widgeon/Hubei/EWHC/2004 (EWHC), A/chicken/Hubei/327/2004 (CKDW), and A/goose/Hubei/ZFE/2004 (GOZF) induced a 100% death rate within 4–13 days, A/chicken/Hubei/XFJ/2004 (CKXF) induced a 50% death rate, and A/duck/Hubei/XFY/2004 (DKXF), A/chicken/Hubei/TMJ/2004 (CKTM), and A/chicken/Hubei/JZJ/2004 (CKJZ) caused no clinical signs or death.

To characterize the antibody responses in mice after infection with avian influenza virus, 6-week-old BALB/c mice were intranasally inoculated with nonlethal doses (CKTM, DKXF, and CKJZ, 10^6^ EID_50_; GOZF, CKDW, CKXF, and EWHC, 10^3^ EID_50_). Serum samples were collected at 0, 15, 30, and 60 d postinfection. Antibody analyses showed that GOZF, DKXF, CKDW, CKXF, and EWHC elicited antibody responses in mice at 30 d postinfection and that the antibody levels increased markedly at 60 d postinfection. In the CKTM and CKJZ groups, however, no antibodies were detected at any point.

The hemagglutinin (HA) and neuraminidase (NA) genes from the isolates were amplified by reverse transcription–PCR (*5*) and sequenced. Phylogenetic analysis was performed with Mega 3.1 software (Evolutionary Function Genomics, Tempe, AZ, USA) (*6*). A neighbor-joining tree was based on the nucleotide sequences of HA and NA genes (Figure 2). For the HA gene, the 6 isolates of higher pathogenicity clustered together and were related to the China Guangdong isolate from wild duck (A/wildduck/Guangdong/314/2004). The HA gene of the less pathogenic DKXF isolate was at a separate position and displayed a close relationship with the Hong Kong goose isolate (A/goose/HongKong/3014.5/2000), which seems to share a common ancestral sequence with the other 6 isolates. For the NA gene, except for CKXF, the other 6 isolates clustered together and were more closely related to several other Chinese isolates obtained in 2004 than to isolates belonging to other strains. The NA gene of CKXF was more closely related to isolates from humans in Indonesia during 2005.

**Figure 2:**
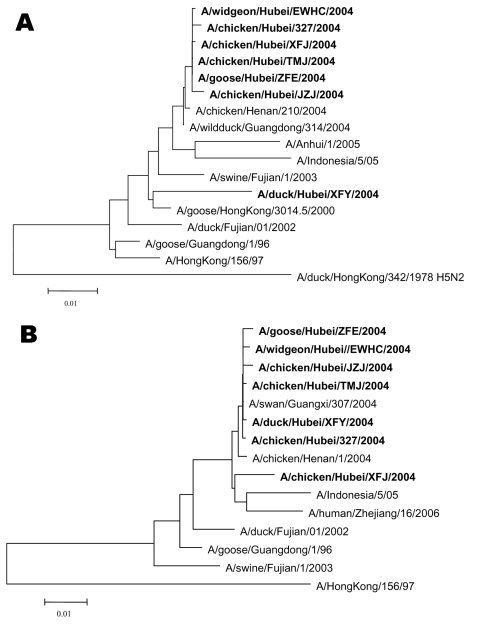
Phylogenetic relationship of various avian influenza virus (H5N1) isolates based on the nucleotide sequences of the A) hemagglutinin and B) neuraminidase genes. **Boldface** indicates strains isolated in this study.

## Conclusions

In this study, influenza virus strains of different pathogenicity were isolated from central People’s Republic of China during the same outbreak. Distinctive from most other studies, our work may lead to a more detailed understanding of the complexity of the genetic and biologic variations among avian influenza (H5N1) isolates from outbreaks. All viruses except DKXF were highly pathogenic to avian species, while their pathogenicity in mice was variable (Table 1). The pathogenicity of EWHC in mice was higher than that of CKDW, but the virus titer in MDCK cells was lower than that of CKDW. The HA and NA gene sequences suggested that these viruses were closely related, but they had different clinical and biologic characteristics. DKXF exhibited low pathogenicity in avian species and mice. These data indicate that the isolated strains had diverse biologic characteristics.

DKXF was highly infectious to chicken eggs and MDCK cells (Table 1) and was found to replicate in chickens and mice despite its lack of pathogenicity. In addition, DKXF could induce high antibody titers in mice and chickens. Moreover, a strong cross-neutralizing reaction between DKXF and CKDW has been recently shown (*7*). Future studies should assess the feasibility of using DKXF as a candidate strain for vaccine development.

Early studies showed that pigeons were more resistant to the highly pathogenic avian influenza (H5N1) strain (A/chicken/Hong Kong/220/97; HK/220) than other birds (*8*). In our experiment, we demonstrated that isolates from 2004 could infect pigeons and could be reisolated from pigeon tissues, especially from glandular stomachs. Sequence analysis indicated that the avian influenza isolates used in this study and the HK/220 strain share high homology at the amino acid level for the HA (95%–97%) but not the NA gene (homology 79%–88%). This study indicates that the pigeon may be an asymptomatic carrier of avian influenza virus.
